# Advances in superhydrophobic material research: from preparation to electrified railway protection

**DOI:** 10.1039/d3ra08180j

**Published:** 2024-04-16

**Authors:** Wang Bo, Zhang Xueqin, Li Bingkun, Liu Yijie, Yang Chenguang, Guo Yujun, Xiao Song, Wei Wenfu, Gao Guoqiang, Wu Guangning

**Affiliations:** a School of Electrical Engineering, Southwest Jiaotong University Chengdu China xq_zhang@swjtu.cn hv-swjtu@163.com; b School of Materials Engineering, Shanghai Jiaotong University Shanghai China

## Abstract

Freezing is a serious problem that affects the power, transport, and transmission industries and is a major concern for the national economy and safety. Currently, several engineering de-icing methods, such as thermal, mechanical, and chemical de-icing, have shown problems related to energy consumption, efficiency, and the environment. Superhydrophobic materials have high droplet contact and roll angles, which can reduce the droplet residence and ice adhesion on their surfaces and have unique advantages in the self-cleaning and anti-icing fields. This paper introduces the development of infiltration theory and superhydrophobic materials and their principles of anti-icing and de-icing. Herein, the preparation and coating methods of superhydrophobic materials in applications are summarised, the performance and lifetime issues of superhydrophobic materials in applications are clarified, and the research progress on superhydrophobic materials in different fields is reviewed. Prospects for the application of superhydrophobic materials in electrified railways are also presented. A feasibility study was conducted to solve some of the existing problems of electrified railways, providing a theoretical basis for the development of electrified railways.

## Abstract 2

Superhydrophobic coatings give a solution for the protection of electrical equipment and some corresponding preparation methods.

## Graphical abstract

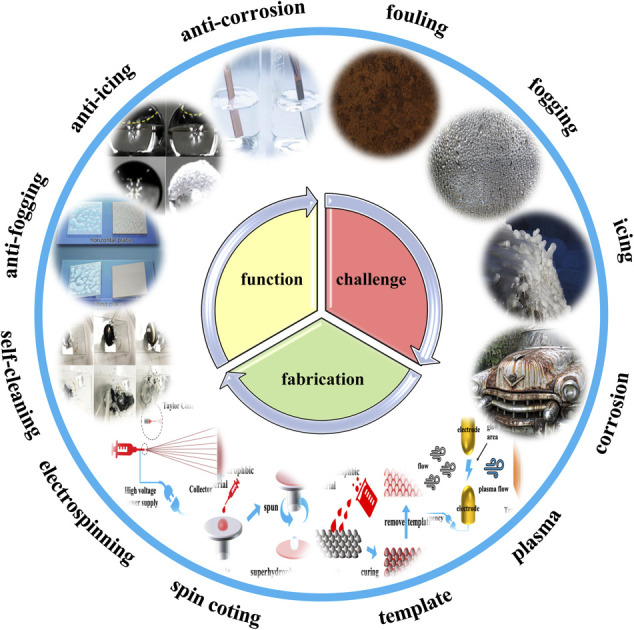

## 1. Introduction

Both water vapor and subcooling water condensation can cause interface ice formation. Ice cover is a common natural phenomenon that threatens the development of national economies and the safety of human life. In the power industry, insulators, lines, and transmission towers are often covered with ice, resulting in the reduction of the surface resistance of insulators and the occurrence of flashover. High gravity loads on lines and towers result in lines breaking and towers falling. In wind power generation, ice-covered motor blades can greatly reduce the efficiency of power generation and shorten the life of electrical equipment. In 2008, the southern part of China and the southeastern part of the United States suffered from ice damage, resulting in the economic losses of RMB 151.65 billion and USD 1 billion, respectively. In the transportation sector, icy roads can increase the incidence of accidents and casualty rates by 84% and 75%, respectively. Ice cover on the catenary can affect the normal flow of high-speed trains. Because of the segmented power supply method of the contact network and weaker conductor strength than the power system transmission lines, thermal ice melting methods in the contact network system have economic and safety restrictions. In 2020, the northeast of China suffered the most powerful ice storm since 1951, resulting in the suspension of 255 passenger trains. Besides, ice on key parts of an aircraft such as the space shuttle will reduce the aircraft's rising force and flight stability and increase flight resistance, which can lead to air crashes. Therefore, the improvement of anti-icing capacity is important for safe and stable operation in electrical, transportation, and other fields. This technology is of great significance to the development and growth of the national economy.

**Scheme 1:**
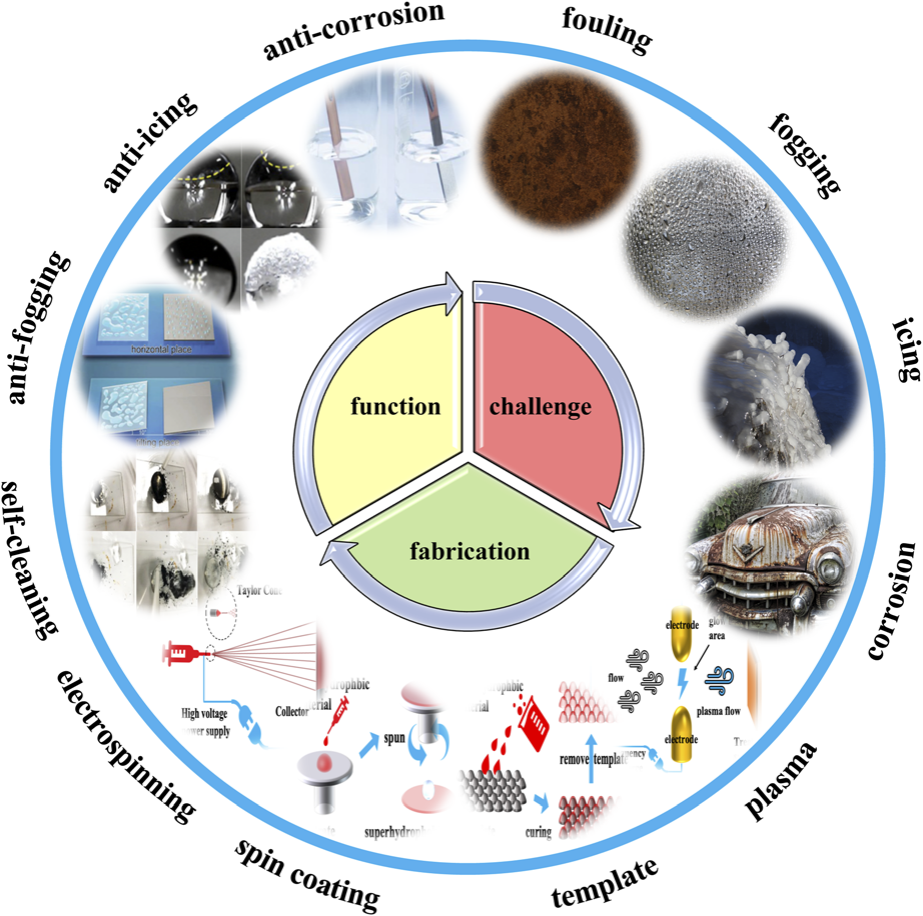
Electrified railways face the challenges of fouling, fogging, icing, corrosion, *etc.* With the increasing availability of manufacturing methods (plasma treatment, stencils, spin coating, electrostatic spinning, *etc.*), various biomimetic superhydrophobic materials have been developed. New functions (self-cleaning, anti-fogging, anti-icing, anti-corrosion, *etc.*) have been given to the interface to solve these problems.

Currently, anti-icing and de-icing means for interface icing problems mainly include human de-icing,^58–60^ chemical de-icing,^61–63^ and thermal de-icing.^64–66^ Manual de-icing consumes a large amount of manpower and is inefficient. It also damages ice-covered surfaces to a certain extent by knocking. Chemical de-icing uses chemicals to lower the melting point of the ice, which can cause the ice to melt quickly. However, the residual chemicals impact the environment. Salts can also corrode metal surfaces. Thermal de-icing is done by heating the ice-covered object, or by warming the ice. This is very energy-intensive, and the melting temperature of the ice-covered object is much higher than the working temperature and can cause thermal deterioration of the ice-covered object. In active anti-icing applications, the high contact angle and low rolling foot of superhydrophobic coatings can greatly reduce the amount of water collected on the surface. The microstructure and nanostructure also delay the icing time of surface droplets. The multifunctional protective properties of superhydrophobic coatings (anti-fouling, anti-fogging, anti-icing, anti-corrosion) are very promising for the protection of equipment in electrified railroads (Scheme 1).

## 2. Wetting theory and de-icing principles

### 2.1. Wetting theory

The concept of contact angle (CA) was introduced by Thomas Young in 1804–1805. It is the angle between the tangent line formed by the interface of the liquid drop and the interface, depicted by Young's equation (the wetting equation) as follows (1):^1^

1 *γ*_LV_ cos *θ* = *γ*_SV_ − *γ*_SL_

**Fig. 1:**
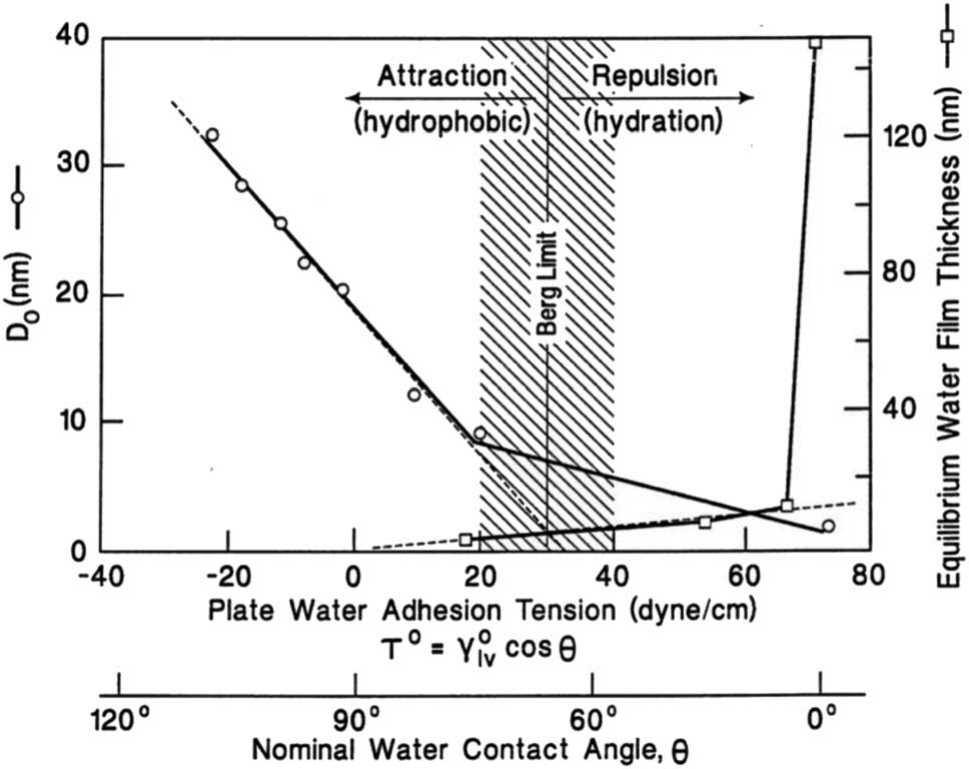
Berg limit based on hydrophobic force measurement. Left-hand axis: monotonic decrease in the characteristic decay length of surface forces with water adhesion tension observed using an atomic force microscope as a surface force apparatus. Right-hand axis: exponential-like increase in condensate film thickness formed from saturated water vapor on quartz surfaces with increasing. Reproduced with permission.^2^ Copyright 1998 Elsevier Science B.V.

*γ*_LV_ is the interfacial tension between liquid and gas. *γ*_SV_ is the interfacial tension between solid and gas. *γ*_SL_ is the interfacial tension between solid and liquid. The CA is related to the surface tension between each of the two phases of the gas–liquid–solid triplet, where *θ* is the apparent CA at the interface. According to this equation, the CA is traditionally defined as 90° for hydrophilic and hydrophobic surfaces. However, after considering the chemical and structural factors of the water droplet. Berg *et al.* confirmed that the defined CA for hydrophilic and hydrophobic surfaces should be 65°.^2^Fig. 1 shows that when the droplet is between two interfaces with CAs greater than 65°, the attachment tension is below 30 dyn cm^−1^ and the droplet shows an inward contraction, indicating a hydrophobic interface. When the droplet is between two interfaces with CAs less than 65°, the attachment tension is above 30 dyn cm^−1^ and the droplet shows an outward tension, indicating a hydrophilic interface. In a later experiment, Jiang *et al.* also confirmed that 65° should be the CA that strictly distinguishes between hydrophilic and hydrophobic surfaces.^3^

The concept of superhydrophobicity was introduced by Reick in 1976 to describe the spherical shape of liquid droplets on the surface of a fumed silica particle coating. On such coatings, the surface adhesion of the droplets was negligibly low. Superhydrophobicity is conventionally defined as an interface with a CA greater than 150° and a roll angle of less than 10°. The superhydrophobic properties are achieved *via* two main factors: a micro–nano scale rough surface structure and low surface energy.

In 1936, Wenzel modified the Young equation (eqn (2)) to explain the wetting theory of rough surfaces. On introducing the concept of roughness, *r*, is defined as the ratio of the actual area of a surface (*S*) to the geometrically projected area (*S*_e_).^4^2
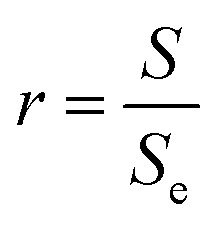
where *S* is the actual surface area and *S*_e_ is the projected area. The Young equation with the introduction of roughness in the one-dimensional regime was modified and the Wenzel equation was proposed (for *r* > 1) as follows in eqn (3).^5^

3 cos *θ* = cos *θ*_e_

where *θ*_e_ is the intrinsic CA of the interface. It follows from the Wenzel equation that an increase in roughness increases the wettability of the surface; in other words, an increase in roughness increases the CA of a theoretical hydrophobic surface (CA greater than 90°) and decreases the CA of a theoretical hydrophilic surface (CA less than 90°).

Considering a non-homogeneous elemental surface, the surface energy per unit area can be calculated by the thermodynamic eqn (4).^6^4
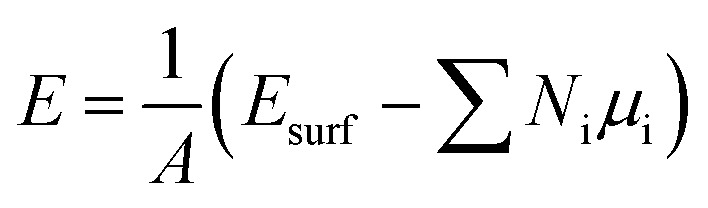
where *E*_surf_ is the sum of surface energy, *A* is the surface area, *N*_i_ and *μ*_i_ are the elemental number and chemical potential energy of element i, respectively. However, the pursuit of a low surface energy cannot achieve a superhydrophobic state at the interface. In a perfluorinated modified plane, the maximum CA is 118°. Lodziana *et al.* prepared θ-Al_2_O_3_ by hydration and despite achieving a negative surface energy can only reach a maximum CA of 120°.^7^

In 1994, Cassie and Baxter *et al.* successively explored the relaxation phenomenon of liquid droplets at interfaces and proposed the effect of the gas–liquid–solid three-phase contact area on CA. They established the Cassie–Baxter equation (followed by (5)) and proposed dynamic CAs (advancing and retreating feet) for water droplets.^8^

5 cos *θ* = ∑*f*_i_ cos *θ*_i_

**Fig. 2:**
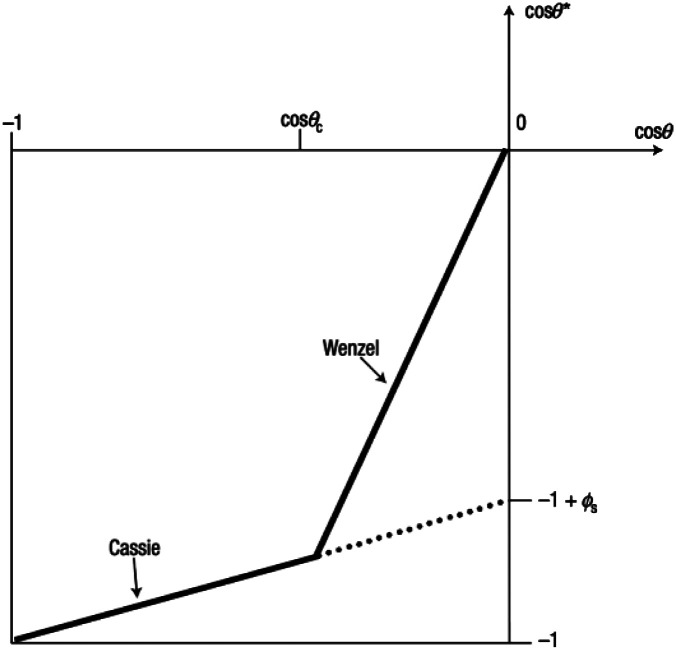
The Cassie and Wenzel regimes of superhydrophobic states: the apparent CA *versus* Young's angle for the hydrophobic surfaces, assuming the roughness remains constant. Roughness makes it possible for the droplet to have more than one metastable position of equilibrium, and the drop can transfer from one metastable equilibrium to the other if the energy barrier can be conquered. The resulting Cassie configuration corresponds to the lowest energy state in the open-air regime, while the Wenzel configuration represents the absolute minimum energy in the wetted hydrophobic state. Reproduced with permission.^9^ Copyright 2003 Nature Publishing Group.

*f*_i_ is the differential area for a CA of *θ*_i_ (∑*f*_i_ = 1). For a single component substance (considering a CA of 180° for air), the Cassie–Baxter equation can be written as eqn (6):

6 cos *θ* = *f* cos *θ*_e_ − (1 − *f*)

where *f* is the contact area as a percentage of the projected contact area. The Cassie state can achieve the single-phase transition of the Wenzel state under the condition of absorbing external energy. The Wenzel equation and the Cassie–Baxter equation can be combined to reveal a threshold cos θ* as eqn (7), represented in Fig. 2:^9^7
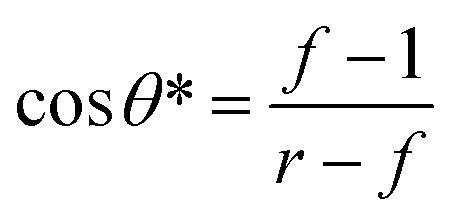


The threshold value is between 0 and −1, at which the Cassie state is likely to change to the Wenzel state.

In the Cassie state, there is more trapped air in the rough structure of the interface. When the Cassie state transitions to the Wenzel state, the water invades the rough structure, the solid–liquid contact area increases, the CA decreases, and the viscosity of the droplet at the interface increases significantly, resulting in the relaxation angle increasing instantaneously by a factor of 10 to 20. This indicates that when the rough structure is invaded. The gas phase in the rough structure condenses into the liquid phase, and the pressure presses the liquid phase into the rough structure. The superhydrophobic surface, therefore, loses its superhydrophobicity, and this state transition is irreversible. As such, we need to design the superhydrophobic surface so that θ* is as small as possible, which gives a larger range of variation in *θ* to keep the whole system in a stable Cassie state. Besides, θ* is related to the surface texture.

In 1997, Barthlott and Neinhuis revealed that the self-cleaning function of the lotus leaf was achieved by the micron-scale papillary projections on the surface (rough structure) and the waxy epidermis on the projections (low surface energy). Through this discovery, they proposed a single-scale microstructural model of the lotus leaf.^10^ In 2002, Jiang found micron-scale papillary projections on the surface of the lotus leaf, and also found that nanoscale dendritic structures were growing on the micron-scale protruding surface of the lotus leaf; this dual-scale microstructure with the coexistence of the microscale and nanoscale was the main factor in achieving the lotus leaf effect.^11^ The team discovered that nanoscale rough structures can achieve superhydrophobic properties by preparing a bionic surface from carbon nanotubes.

With the continuous improvement of the wetting theory, superhydrophobic materials are gradually being realized for engineering applications. The applications in different fields have brought to light the problem of the environmental erosion resistance of superhydrophobic materials. Currently, there are three ways to solve this problem: (1) self-healing surfaces, in a bionic way, which is complex and expensive;^12^ (2) flexible surfaces can disperse the pressure on the surface through elastic deformation and restore the surface structure after the pressure is withdrawn;^13^ (3) multilayer self-similar structures, where the newly created surface remains superhydrophobic after the outermost surface is damaged.^14^ In addition to this, in 2020, Deng *et al.* equipped fragile surface structures with high-strength ceramic armor, making a landmark new development in the problem of wear and corrosion resistance of superhydrophobic materials.^15^

### 2.2. De-icing principles

**Fig. 3:**
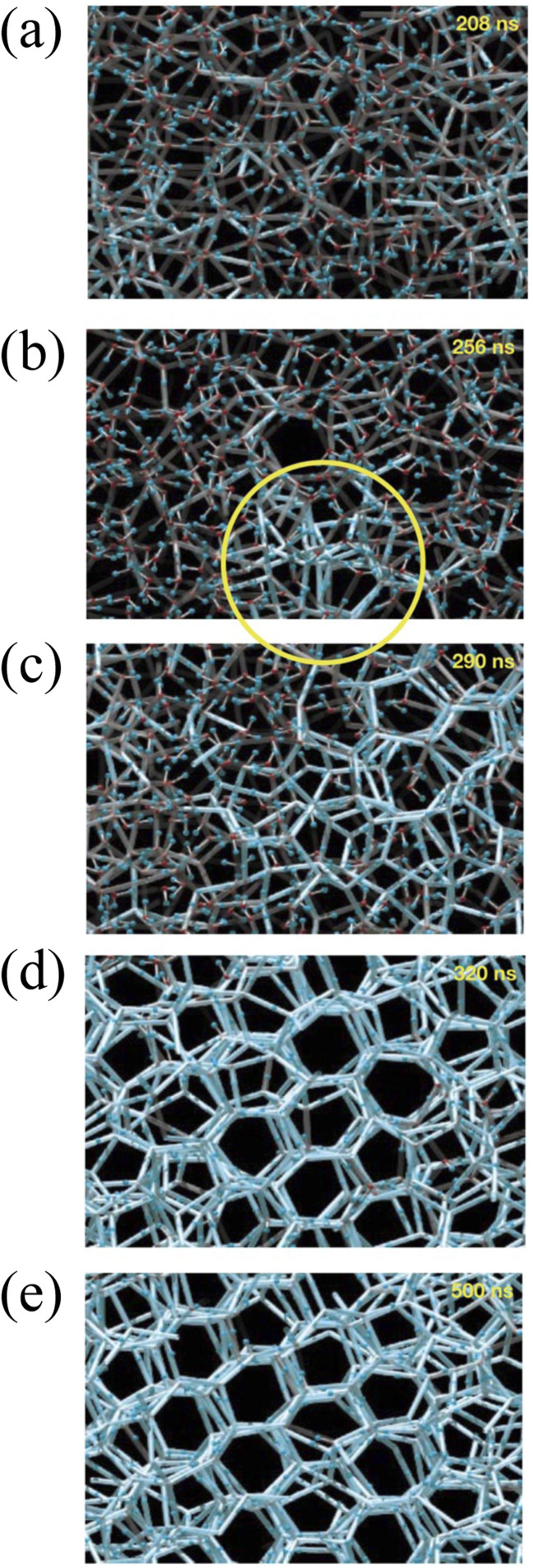
The hydrogen bond network structure of water at a given time in inherent structures. (a) At time *t* = 208 ns; (b) at *t* = 256 ns; (c) at *t* = 290 ns; (d) at *t* = 320 ns; (e) at *t* = 500 ns. Lines indicate hydrogen bonds in water molecules, and the intermolecular bonds of water participating in such hydrogen bonding. Bright blue lines indicate ‘long-lasting’ hydrogen bonds, which have persisted longer than a specified threshold value before a given time *t*. The brightest blue lines are those with a lifetime longer than 2 ns (*τ*_life_ > 2 ns). An initial nucleus is formed in the region circled in (b). Reproduced with permission.^16^ Copyright 2002 Nature Publishing Group.

The process of water icing at an interface is divided into five stages: liquid subcooling, nucleation, re-glow, freezing, and solid cooling. Masakazu *et al.* simulated the various processes of water icing by molecular dynamics as shown in Fig. 3: in the early part of the liquid subcooling stage, the network of hydrogen bonds in the water exists in the form of 5 to 7 rings of water molecules linked by hydrogen bonds (5 to 7 membered rings); however, this structure constantly breaks down and reorganizes. The blue hydrogen bonds in the diagram indicate long-lived hydrogen bonds with a lifetime of more than 2 ns (average lifetime of 1 ps at *T* = 300 K and 180 ps at *T* = 230 K). The long-lived hydrogen bonds occur randomly at different locations. At *t* ≈ 256 ns, a polyhedron of long-lived hydrogen bonds forms and slowly grows by changing its position and shape through changes in the surrounding hydrogen bonds. It is eventually pinned in a definite position at *t* ≈ 290 ns. The ice nucleus then grows rapidly throughout the 3D space by transforming the surrounding hydrogen bonding network into a six-membered ring, while reducing the overall potential energy. At the end of the rapid growth, a stacked honeycomb structure of six-membered rings is formed throughout the system. The final freezing process is an extremely slow process that is accompanied by a further reduction in the overall potential energy.^16^

The condition for nucleation in a hydrogen-bonded network is the overcoming of the potential barrier (Gibbs free energy difference,^17^ Δ*G*_c_ as shown in (8)):8
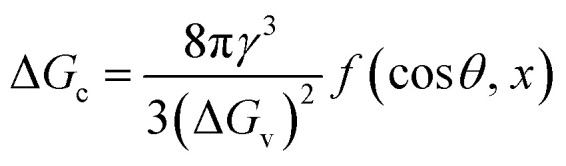
where *f*(cos *θ*, *x*) is as follows:9
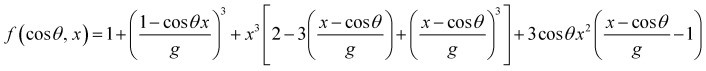


*x*, *r*_c_, and *g* are given as follows in eqn (10)–(12):10
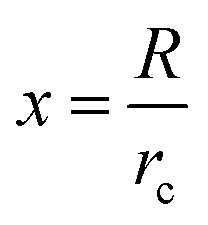
11
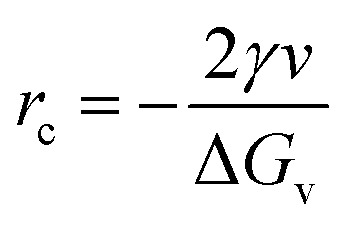
12
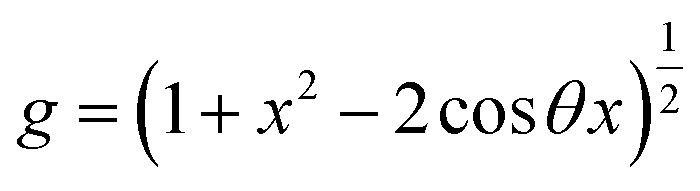
where *γ* is the interfacial tension between the two phases. *r*_c_ is the critical nucleation radius (the minimum radius that ice crystals need to reach to maintain a stable crystallization process, and ice crystals will only grow at the surface if their size is larger than the critical nucleation radius). *v* is the molar volume of water, and *G*_v_ is the difference in free energy per unit volume between the solid and liquid phases of water.

Using the above equations, it is easy to obtain the overcoming potential and the critical nucleation radius required for ice cover. The principles of anti-icing and de-icing on the surface of superhydrophobic materials are divided into the following three points: (1) prolonging the overcooling phase; (2) reducing the droplet surface residence time. (3) Reducing the surface water collection.

During the overcooling phase, the air trapped on the surface of the superhydrophobic material reduces the actual solid–liquid contact area, thus, significantly reducing the efficiency of heat transfer between the solid and liquid and ultimately, extending the duration of the overcooling phase. Studies have shown that a superhydrophobic surface can prolong the time for droplets to freeze on the surface if the roughness of the surface *r* ≦ *r*_c_.^18^

If the adhesion of water droplets on superhydrophobic surfaces is low, it is easy for droplets to roll off from their surfaces at low adhesion. The Young–Dupre equation shows that droplet roll-off on a superhydrophobic surface requires overcoming the adhesion work *W*_a_(13):

13 *W*_a_ = *γ*(1 + cos *θ*)

Thus, the CA of a superhydrophobic material is positively related to the adhesion work of its surface droplets. C. Antonini accurately reconstructed the contact line shape and different CA distributions of droplets with the surface through multiple profile images of water droplets. The adhesion force of irregular droplets at the surface was accurately calculated with an error of 1%. It shows that the adhesion force is determined by the CA, contact line shape, length, and surface tension together.^19^ Pierce *et al.*, Ha *et al.*, and Bertolucci *et al.* showed that the roll-off angle of a droplet at a surface is related to the roughness of the surface and the inhomogeneous distribution of the chemical composition. In addition to the interfacial contact force, an increase in the *r*-value increases the surface relaxation angle, leading to an increase in the roll-off foot.^20–22^ This in turn forms a reciprocal constraint with the previous statement that increasing *r* values favour delayed icing of the surface. Therefore, it cannot consider only one-sided parameters in the design of anti-icing surfaces using superhydrophobicity.

During dynamic droplet impact on the surface (rain falling freely on the surface or during transport), droplets on the superhydrophobic surface undergo a “pie-like bounce”, a process of flattening to coalescence and then bouncing. This process reduces the contact time between the droplet and the surface and reduces the amount of water collected at the surface, which may be washed away from the surface by the surrounding flow field in the process. This process is accompanied by two physical parameters: the bounce time and the bounce conditions. A study by Richard *et al.* showed that the bounce time *t*_0_ of a droplet on a surface followed by (14) is related to the droplet density *ρ*_0_, the droplet radius, *R*_0_ and the liquid–gas surface tension *γ*_LV_:^23^14
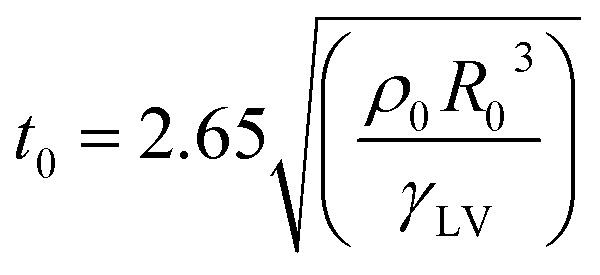
When *t*_0_ is less than the freezing time, the droplet is unable to freeze on the surface. Another parameter, the bounce energy, was shown in Reyssat *et al.* to occur when the kinetic energy of a droplet is greater than its diffusion storage energy (15):^24^

15 *ρ*_0_*R*_0_^3^*v*_0_^2^ > *γ*_LV_*R*_0_^2^Δcos *θ*

where *v*_0_ is the velocity of the droplet's impact. It can be seen from the equation that the larger the droplet diameter, the greater the velocity, the smaller the CA relaxation, and the more likely it is that bouncing will occur. However, as mentioned earlier, water droplets with velocity and external pressure tend to change from the Cassie state to the Wenzel state pinned at the surface, resulting in the failing of the anti-icing function. Therefore, the design of a superhydrophobic surface for practical anti-icing functions must also minimize the CA relaxation.

## 3. Preparation methods

Over millions of years, organisms in nature have evolved a variety of complex features to better adapt to their environment. These include superhydrophobic structures on the surfaces of living organisms. The most typical of these is the lotus leaf, which achieves a CA of 160° through its micro-papillae, nano-dendritic structures, and waxy surface with low surface energy; the leaf structure of rice is similar to that of the lotus leaf in that both have papillary rough structures on the surface. The difference is that the papillae on rice leaves show a quasi-one-dimensional linear arrangement along the direction of the petiole, which makes the rolling feet on the starting surface anisotropic: the angle of roll is 3° to 5° along the petiole direction and 9° to 15° in the vertical petiole direction; the butterfly wings, achieve a rolling foot of approximately 9° in the outward radiation direction of the wings through the orderly stacking of lamellar micro–nanostructures, while in the opposite direction the water droplets can be pinned in a 90° plane. The legs of the water strider are long with directionally arranged needle-like micro-hairs and spiral patterns. With the structure at an angle of 20, it can effectively trap air on the water surface without piercing the surface within 4 mm, and the repulsive force of the air trapped by a single leg against the water surface is sufficient to support 15 times the weight of the water strider. In addition to the above-mentioned plants and animals, there are also acacia leaf pinks and desert beetles that combine both hydrophilic and hydrophobic wettability in their application. All of them are perfect examples of the survival of the fittest. As human observation methods and preparation methods continue to advance, various methods of preparing biomimetic superhydrophobic materials have been realized in the process of learning from nature: plasma treatment, spin coating, spraying, electrostatic spinning, sol–gel, hydrothermal, electrochemical, laser etching, chemical vapor deposition, *etc.*

### 3.1. Plasma treatment

**Fig. 4:**
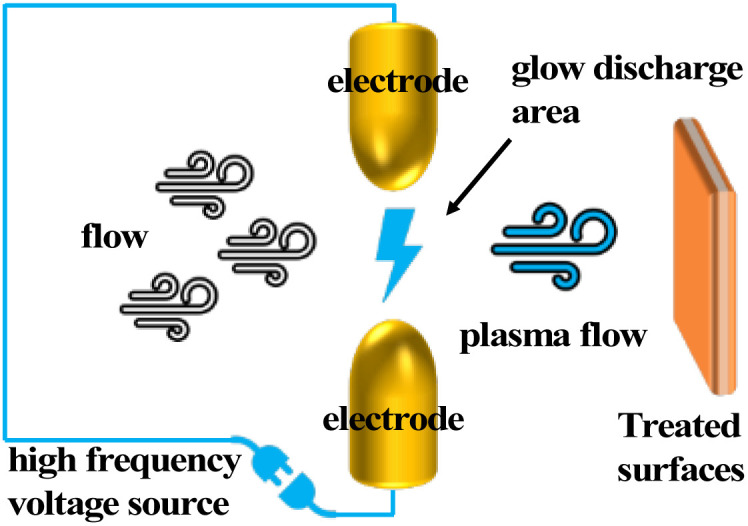
Schematic diagram of plasma treatment.

Plasma surface modification technology has a wide range of applications in the field of materials. The addition of an etchant to the ion stream significantly improves the etching efficiency as compared to the commonly used etching method of argon ion bombardment of the surface. The etching radicals in the etching ionomer stream diffuse to the surface of the material where adsorption occurs. Reaction with the material occurs to change the material surface properties and morphology. The gaseous by-products generated during the reaction are desorbed by ion bombardment. The plasma formed differs depending on the discharge principle. In addition, the discharge environment, discharge power, *etc.*, also have an impact on the formed plasma, while the distribution and intensity of the plasma have a direct effect on the surface state, and the effect of different treatment times on the surface state such as surface cleaning and etching during the treatment process is also different (Fig. 4).

Christopher S. Yung found that changing the components of the plasma stream could change the wettability of the carbon nanotube surface by plasma treatment of vertically aligned carbon nanotubes. When the plasma stream was oxygen, the treated carbon nanotube walls embodied superhydrophilicity; after adding CF_4_ to the plasma stream components, the contact angle of the carbon nanotube walls reached 159°.^25^ In addition to high-pressure plasma treatment methods, atmospheric and low-pressure plasma treatments are also suitable for the preparation of superhydrophobic surfaces. E. Vazirinasab developed a simple, environmentally friendly, and industrially suitable method for the preparation of superhydrophobic surfaces based on an atmospheric pressure air plasma system. By this method, microstructured and nanostructured surface roughness was established on high-temperature vulcanized (HTV) silicone rubber surfaces, and the superhydrophobic surfaces obtained had static water contact angles >160° and contact angle hysteresis <3°. By comparing the work of others, it was found that superhydrophobic surfaces were more easily prepared when the reference voltage was 90–100% and the plasma jet speed was 4 m min^−1^.^26^ Gurusamy Shanmugavelayutham treated the textile with low-pressure plasma and the fabric surface possessed anti-ice covering properties.^27^

The plasma treatment method is highly effective in the preparation of superhydrophobic surfaces. The process is simple and environmentally friendly, with precise control of the treated surface properties through process parameter adjustment. The treatment results do not affect the overall material properties. The disadvantage is that the lifetime of the treatment effect declines with time and cannot be effective in the long term.

### 3.2. Template

**Fig. 5:**
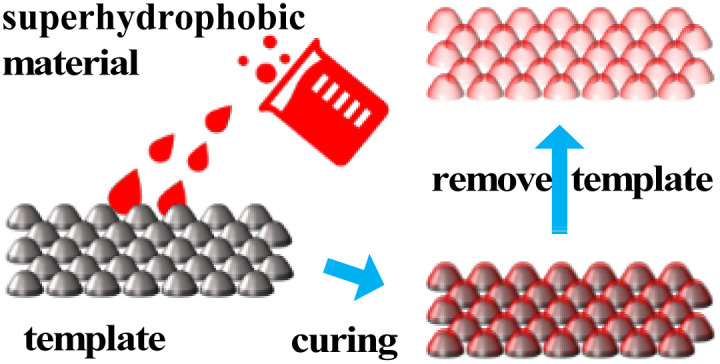
Schematic diagram of template.

The template method involves using a material with a rough surface or pore structure as a template and preparing the film-forming solution on the template by coating, casting, or deposition, then removing the template to form a superhydrophobic surface on the surface of the film (Fig. 5).

Usually, superhydrophobic surfaces can be achieved by precisely tuning the size of the stencil to achieve surface microstructure by the stencil method. Simonetta Rima used cellulose as a template to prepare silicon oxide fibers and adjusted the concentration of ethyl orthosilicate to control the fiber thickness. The fibers were deposited onto the surface of the particle substrate and surface modification was performed on the fibers, resulting in a surface with a static contact angle of approximately 159°.^28^

Using the superhydrophobic surface itself to prepare custom surface stencils with differential wettability for use in microfluidics or chips is also an alternative approach. Lai used inkjet printing to print hydrophilic materials onto PDMS substrates for economical and fast microfluidic control of surfaces.^29^ Sun converted the substrate and inkjet wettability to achieve similar functionality as well. Such stencils, prepared using surfaces with a customized distribution of wettability, have great potential for material design, device fabrication, and interface studies.^30^

The template method for the preparation of superhydrophobic materials allows the material and structure of the template to be designed according to the properties and form of the material. For some materials that are difficult to form in one go, the template can be designed several times. However, the template method is limited to small areas in the laboratory and cannot be produced on a large scale. Besides, the template needs to be removed by physical or chemical processing after the material has been prepared.

### 3.3. Spin coating or spraying

**Fig. 6:**
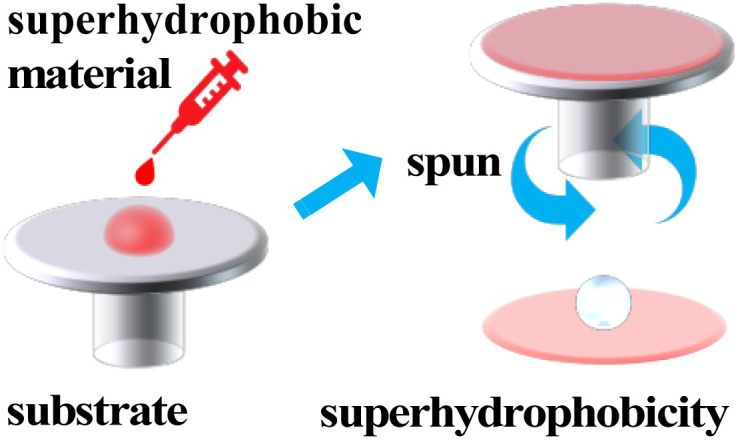
Schematic diagram of spin coating.

Spin coating is a method where the spin-coating fluid is applied dropwise to the surface of the substrate and spun with high speed to spread it onto the substrate to form a homogeneous film, then dried to remove the remaining solvent and obtain a film with stable properties (Fig. 6).

Tan used a simple spin-coating method with methyl MQ silicone resin (Me-MQ) modified SiO_2_ nanoparticles and silane coupling agents (KH-570). Eventually, a fluorine-free superhydrophobic coating with significant superhydrophobic properties with a maximum CA of 168.8° and a minimum sliding angle of 1.0° (ref. 31) was obtained.

Zhai changed the monolayer distribution of gold nanoparticles on the surface by spin-coating. The monolayer distribution of gold nanoparticles was transferred to a PDMS surface with a rough structure by vacuum hot pressing, resulting in a superhydrophobic surface. The transferred gold nanoparticles were found to have a higher contact angle on the surface. It was also found that the surface with gold nanoparticles could more easily keep the droplet in the karstic state as the droplet volume decreased.^32^

Xin prepared organic–inorganic PVDF/PVDF–SiO_2_ hybrid matrix membrane contactors by using a hydrophobic modification method. The suspension was introduced onto the PVDF substrate surface using spin coating. Partial etching of said PVDF particles was used to construct an adhesive PVDF–SiO_2_ core–shell layer on the PVDF substrate, resulting in a more stable PVDF–SiO_2_ coating. The complete porous PVDF–SiO_2_ layer was formed by varying the etching morphology of the PVDF particles and the amount of doped PVDF and SiO_2_ particles. The resulting PVDF/PVDF–SiO_2_ film contactor had a more regular pore size distribution, excellent hydrophobic properties, and a water CA close to 158°.^33^

Emel Yilgör compared the effects of both scratch-coating and spin-coating preparation methods on the surface roughness and hydrophobic properties of the coatings. It was found that the number of layers of spin coating had almost no effect on the coating and was a stable preparation method.^34^

For the time being, the spin coating method can be adequate and uniform in the preparation of small-sized films, but it has not been possible to achieve large-area coating; this works only in the laboratory. The thickness of the coating is uniform, and it is difficult to control as the spin coating area increases.

Zhang used the spraying method to prepare edible superhydrophobic coatings for food packaging. They successfully prepared a superhydrophobic surface by heating and mixing coffee lignin and beeswax in a certain ratio and then spraying the mixture onto the substrate using a spraying machine. The coating exhibited high water resistance to a variety of liquids, with CAs all above 150°.^35^

The spraying method can be applied to a large area under stable conditions. Regardless of the shape and size of the specimen, the spraying effect is highly dependent on the operator's technique.

### 3.4. Electrostatic spinning

**Fig. 7:**
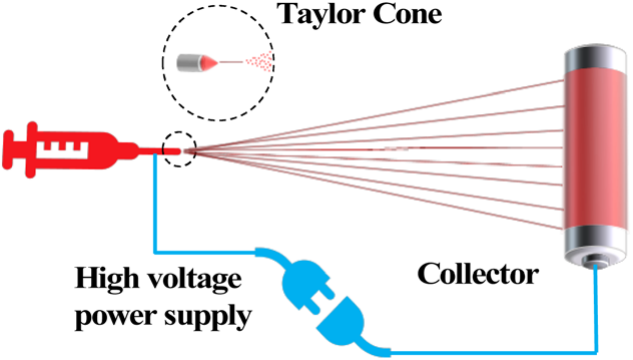
Schematic diagram of electrostatic spinning.

The concept of electrostatic spinning was introduced by Zeleny in 1917, and the first polymer fibers supported by this technology appeared in 1934. In the 1990s, the technology attracted much attention and has advanced significantly in the 21st century. The electrostatic spinning method is considered to be a type of electrostatic atomization. It is also known as electrospinning, in which an externally reinforced electric field causes a Taylor cone jet stream of polymer solution or melt to form at the jet orifice while stretching and volatilizing in an electrostatic field environment to form fibers cured on a receiver plate. Nearly one hundred kinds of natural macromolecules and synthetic polymers have been prepared into nanofibers by electrostatic spinning technology. They are used in many fields such as biomedicine, catalysis, engineering microcarriers, aerospace optoelectronic devices, and so on (Fig. 7).

Yu prepared an asymmetric medical dressing by electrospinning. The hydrophilic side can promote wound healing to the inside and the hydrophobic side has a waterproof function to the outside.^36^

Jiang's film showed a microsphere and fiber stacking effect by controlling the humidity at a high level during the electrospinning process; it had excellent breathability while being super hydrophobic.^37^ Cheng prepared superhydrophobic films with interwoven fiber microspheres by coaxially blending PVDF and PDMS with different concentrations by the asymmetric electrospinning method. Highly effective water–oil separation was achieved.^38^

By coupling spinning technology with hot pressing technology, Juliana collected PLC on LDPE films by spinning. The SiO_2_ nanoparticles were then collected by electrospinning on the PLC and finally, the multilayer films were integrated by hot pressing for one minute. Tightly packed superhydrophobic films were prepared.^39^

Li's invention of centrifugal spinning technology also realized the electrostatic spinning of the preparation of the thin film effect. Compared with electrostatic spinning, centrifugal spinning technology is simple and inexpensive to operate and does not require the use of a high-voltage power supply, which is safer.^40^

This method of electrostatic spinning has demanding requirements on parameters such as preparation temperature, humidity, solution viscosity, working voltage, and receiving distance. The coordination between the parameters is extremely important, making it more difficult to prepare. It is also faced with problems such as low mechanical strength and mass production.

### 3.5. Sol–gel

The sol–gel method generally involves the hydrolysis of precursors under acid or alkali conditions to produce active hydroxyl groups. This is followed by a hydrolytic condensation reaction to form a sol, which further increases in viscosity as the hydrolytic condensation reaction proceeds. The sol–gel method can be used to construct rough surfaces for the preparation of superhydrophobic materials. The sol–gel method is one of the methods for the preparation of organic/inorganic hybrid materials because the reaction conditions are mild and can be performed at room temperature and pressure.

Maleki used the sol–gel method to prepare superhydrophobic/superoleophilic PMSQ–SF IPN hybrid aerogels with a graded structure and lightweight compressible mesopores, using 5-(trimethoxysilyl)pentanoic acid (TMSPA), silk protein (SF), and polymethylsilsesquioxane (PMSQ) as raw materials.^41^

E. Vazirinasab incorporated nanosilicon oxide and micron aluminum trihydrate in high-temperature vulcanized silicone rubber, and a superhydrophobic silicone rubber was prepared; the superhydrophobic silicone rubber was both anti-icing and abrasion-resistant.^42^

Wang used the surface energy difference between the fabricated binary-filled phases to achieve spontaneous wrapping agglomeration between the filled phases. The conventional PDMS/SiO_2_ superhydrophobic coatings were modified using h-BN. The prepared superhydrophobic coatings showed improvement in stability, electrical properties, and hydrophobicity.^43^

The sol–gel method has the advantages of low cost, simple operation, and mild experimental conditions in the preparation of superhydrophobic materials, however, the fabrication time is long. Most of the reactants used are organic compounds, which are non-environmentally friendly, and the prepared products are relatively easy to crack.

### 3.6. Hydrothermal

The hydrothermal method involves preparing materials in a sealed pressure vessel, using water as a solvent. The powder is dissolved and recrystallized. Compared to other powder preparation methods, the powder produced by the hydrothermal method has the advantages of complete grain development, small size, uniform distribution, and lighter agglomeration of particles. Cheaper raw materials can be used, and a suitable stoichiometry and crystal shape can be obtained easily.

**Fig. 8:**
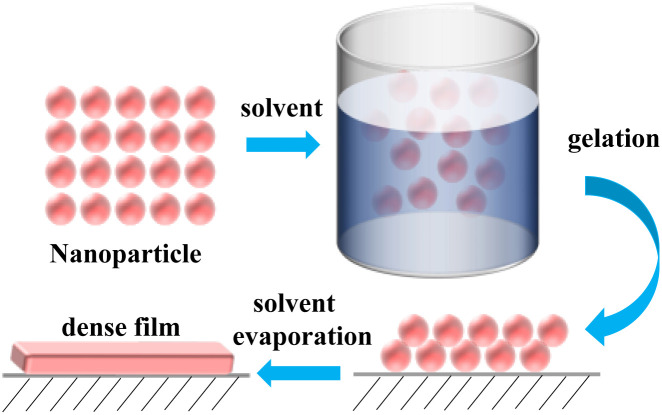
Schematic diagram of the sol–gel method.

**Fig. 9:**
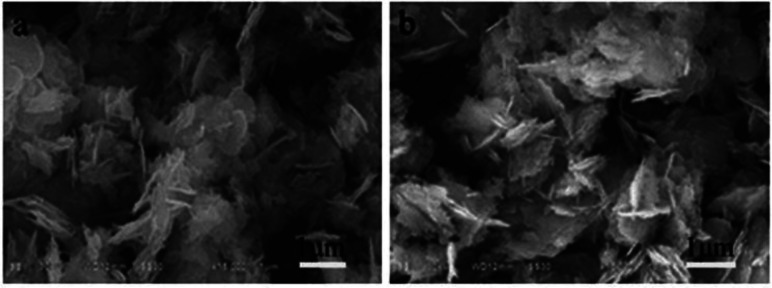
SEM images of BTW1 synthesized in (a) 24 h, and (b) 48 h. Reproduced with permission.^44^ Copyright Springer.

Yang synthesized a series of Bi_2_WO_6_/TiO_2_ impurity junctions by the hydrothermal method. The crystalline phase and morphology were controlled by controlling the amount of modifier, reaction time (Fig. 8), and reaction temperature (Fig. 9). The heterojunctions obtained under suitable reaction conditions exhibited remarkable superhydrophobic properties.^44^

Zhang grafted a large amount of –CH_3_ and –CF_3_ on the surface of an aluminum sheet by the hydrothermal method, which gave the sheet anti-corrosion and self-cleaning properties with a contact angle of 167.2°.^45^

The advantages of the hydrothermal method for the preparation of nanomaterials have made it popular with researchers. However, the reaction environment of the hydrothermal method is under high temperature and pressure conditions. It is slightly less safe and also cannot be produced on a large industrial scale; besides, its energy consumption is relatively high.

### 3.7. Electrochemical

The electrochemical method uses the order of activity of different metallic elements to construct microstructures and nanostructures by allowing substances to be deposited onto the cathode or to corrode the anode in the presence of an electric current. The method was first applied to prevent the electrochemical corrosion of metals, *i.e.*, to protect the cathode material.

Nakayama used a simple electrochemical method, in which superhydrophobic CeO_2_ was deposited on the surface of clean 304 stainless steel. Initially, after the deposition was completed, the coating appeared hydrophilic. After exposure to air, the coating trapped the C and H compounds in the air and exhibited superhydrophobic properties. The coating also had a certain self-healing effect.^46^

**Fig. 10:**
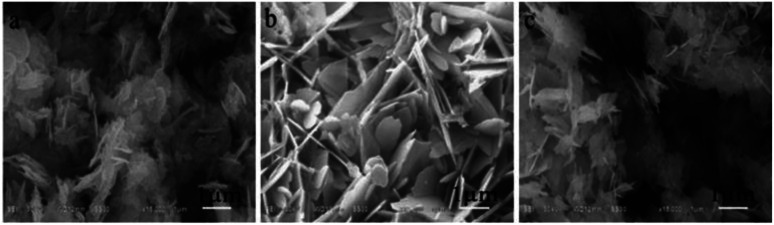
SEM images of the BTW1 sample synthesized at (a) 140 °C, (b) 160 °C, and (c) 180 °C. Reproduced with permission.^44^ Copyright by Springer.

By combining electrochemical with template methods, Chen prepared metal–organic framework (MOF) films with easy peel-and-transfer *via* electrochemical printing on superhydrophobic micro-pillar structure substrates (Fig. 10a). Optical images of the top surface (Fig. 10b) and cut surface (Fig. 10c) of the films show that the method enabled the self-assembly of ZIF spherical particles into MOF films. This has improved the productivity and the application of this type of film in various fields.^47^

Although the electrochemical treatment is better, it consumes more energy. Among other things, electrodeposited nanocrystals have many excellent properties as compared to ordinary crystals, such as corrosion resistance, hardness, wear resistance, ductility, electrical resistance, electrochemical properties, and catalytic activity, which make it promising for a wide range of scientific, technological, and industrial applications.

### 3.8. Laser etching

High-power pulsed laser beams converge on the surface of the material, which can generate high temperature and high pressure in an instant to modify the properties and morphology of the material surface. In recent years, the preparation of micro and nanostructures by laser ablation has become a very important development in the field of new materials research. This technique possesses the property of ultra-fast light–matter interaction. Since the reactor wall is not involved in the whole reaction, there is no pollution of the product. The root preparation environment can be divided into the gas phase and liquid phase, which are used for the preparation of nanomaterials and material surface modification, respectively.

**Fig. 11:**
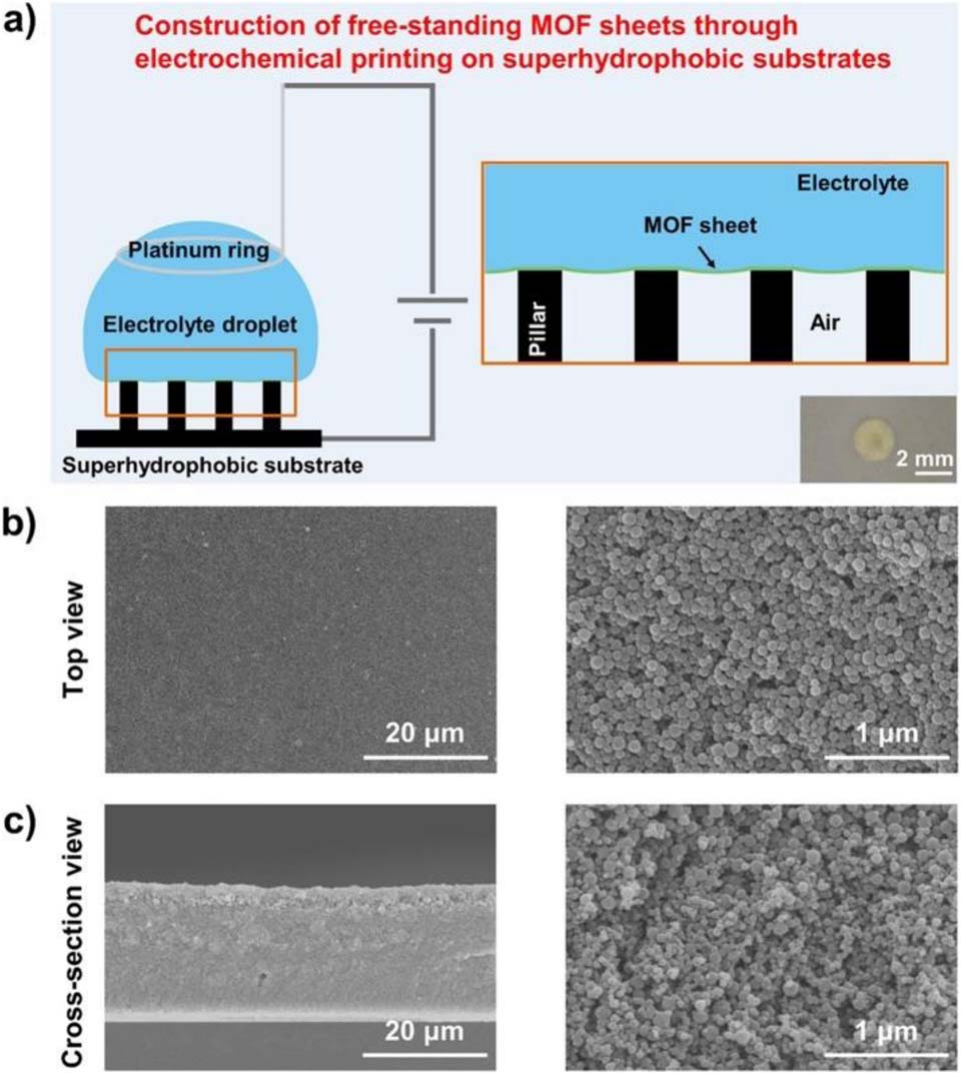
The construction of free-standing metal–organic framework sheets through electrochemical printing on superhydrophobic substrates. (a) Schematic illustration of the construction of free-standing MOF sheets along the solid/liquid/gas triphase interface through the experimental setup for electrochemical printing, which includes a platinum ring, an electrolyte droplet, a superhydrophobic micropillar-structured substrate, as well as a power source. As shown in the brown box, the electrophoretic deposition will occur along the triphase interface consisting of the working electrode, air, and the electrolyte droplet when the experimental setup is powered on. The optical image in the lower right corner shows that the obtained MOF (*i.e.*, ZIF-8) sheet exhibits a nearly circular shape. Scanning electron microscopy (SEM) images of the sheet (left) and the enlarged image (right) from the top (b), and the cross-section views (c), which consist of spherical ZIF-8 nanoparticles stacked together. Reproduced with permission.^47^ Copyright American Chemical Society.

Chen confined a 355 nm UV laser between a fluorinated ethylene propylene film and a polyimide film. Fluorine covalent laser-induced graphene with stable superhydrophobic properties was prepared. Its superhydrophobicity is, on the one hand, due to the low surface energy brought by the fluorine reference. More importantly, it is a laser-constructed microsurface structure. This can be seen in Fig. 11. By varying the laser processing parameters, *e.g.* power, scanning speed, spacing, and defocusing distance, the modulation of film resistance and wettability can be realized.^48^

Laser-induced graphene was used by Sujit Deshmukh by combining a bias DC of 2 V and different voltage polarities, and the droplet was made to undergo a mutation from the Wenzel state to the Cassie state on the film. The directional transport of droplets on the surface was achieved.^49^

Laser etching has superior characteristics such as no contact, high flexibility, fast processing speed, no noise, small heat-affected zone, and very small spots that can be focused to the laser wavelength level, which can achieve good dimensional accuracy and processing quality. It is, however, more costly and less economical.

### 3.9. Chemical deposition

Chemical deposition takes place during a chemical reaction in which the product self-assembles and is deposited on a suitable substrate. The method is adapted to the preparation of thin films of crystalline inorganic materials such as ZnS, CuSe, InS, CdS, *etc.* Chemical deposition methods include chemical bath deposition (CBD), chemical vapor deposition (CVD), and electrochemical deposition (ECD). Depending on the material and deposition conditions, different surface profiles can be obtained, such as nanoneedles, nanotubes, nanorods, *etc.*

**Fig. 12:**
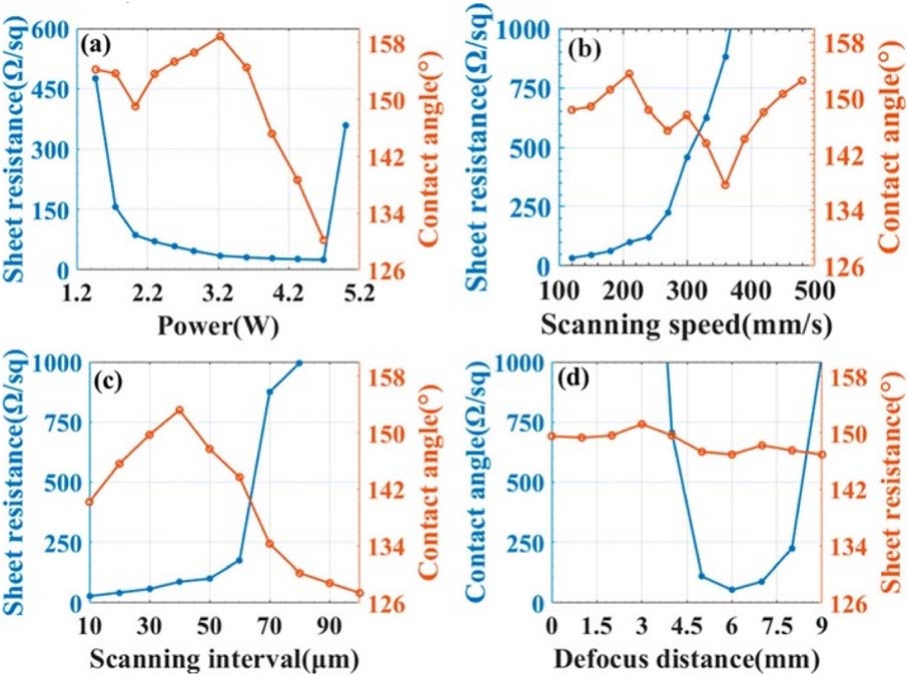
The effects of laser processing parameters (a) laser power, (b) scanning speed, (c) scanning interval, and (d) defocusing distance on the sheet resistance and contact angle of films. Reproduced with permission.^48^ Copyright American Chemical Society.

By comparing the superhydrophobic coatings produced by different preparation methods, Norbert J. Janowicz showed that the aerogel-assisted vapor deposition (AACVD) method (shown in Fig. 12) can reduce the mass fraction of the filled phase from 41 wt% to 9 wt%.^50^

**Fig. 13:**
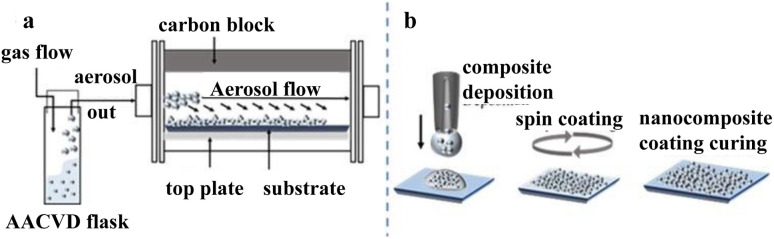
Schematic illustrations of our (a) AACVD and (b) spin coating procedures. Reproduced with permission.^50^ Copyright American Chemical Society.

Tan prepared a highly crystalline “litchi” structure of low-density isotropic pyrolytic carbon by chemical vapor deposition. The coated surface was highly durable and superhydrophobic and had good biocompatibility (Fig. 13).^51^

The use of chemical deposition allows the production of functional coatings such as wear, corrosion, oxidation, and erosion resistance. However, the chemical vapor deposition process is difficult to control, and multiple materials react with each other and may generate new by-products.

At the same time, due to the high reaction temperature, it is very easy to cause the deformation of parts and organizational changes on the material surface. In practical application, the parameters must be controlled scientifically and reasonably. The thermodynamic study must be reinforced to ensure that the prepared material quality is reasonable and demonstrates excellent performance.

Different preparation methods can ultimately achieve a material surface with superhydrophobic properties. However, for the electrified railway method for the surface modification of equipment, three factors still need to be considered: economy, efficiency, and safety. For the diversity of electrical equipment in railroads, a single method is not destined to satisfy all the factors. Therefore, it is necessary to combine several methods to accomplish the efficient modification of the equipment surface.

## 4. Applications of superhydrophobic materials

With the development of the electrified railroad, high-speed railroad transportation plays an important role in national travel and economic development. The high-speed railroad system traces back to the source of the power grid power generation, power transformation, power transmission, traction substation, and to the train by the flow of operation, and interlocking, which in addition to the problem will affect the safe and stable operation of the entire system. These link the design industry, and the wide range from electrical engineering to civil engineering, and from insulation to structural design, needs to be considered. Among the considerations, the protection of equipment is the most important.

### 4.1. The protection of electrical grids and electrified railroads

The grid consists mainly of power plants, transmission grids, and distribution grids. The transmission grid is the power delivery network that connects power plants and substations, or substations and substations. It is mainly responsible for the task of delivering electricity. The distribution network receives power from the transmission network or regional power plants. Distribution is facilitated through local distribution or by voltage step-by-step distribution to various types of users of the power network. It is composed of overhead lines, cables, towers, distribution transformers, disconnect switches, reactive power compensators, and some ancillary facilities. It plays an important role in distributing electric energy in the power network. A huge amount of power is transmitted through high-voltage lines. Overhead transmission grids are suitable for long-distance transmission of electricity. Underground transmission grids are used only in densely populated areas. Cables are used when overhead lines are not suitable for that application situation. Underground cables come with insulation. To prevent leakage of electricity from the power supply, transformer equipment, and electrical appliances, and to avoid the accumulation of charge in the metal casing, the cables are provided with a protective device – ground. Electricity for electrified railroads or urban electric traffic is supplied by traction substations. The main power equipment is a step-down transformer (called the main transformer or traction transformer) with a single capacity of more than 10 000 kVA.

Most of the power equipment used in power generation, transmission, and distribution are operated in the open air. They are exposed to wind, sand, rain, snow, and corrosion. In the whole power system and electrified railroad operation process, the corrosion of the metal structure will lead to the degradation of the support structure strength, the outer insulation surface of the dirt and liquid adhesion lead to the deterioration of the electrical strength, the contact network overlaying ice leads to the interruption of the bow network first-flow system, and other problems seriously affect the normal and safe operation of the electric power and electrified railroad system. The multifunctionality of superhydrophobic materials has great application prospects in the protection of electric power systems.

### 4.2. Anti-corrosion

Superhydrophobic materials can greatly reduce the contact area between the surface and corrosive liquids, thus achieving the function of surface corrosion prevention. Surface corrosion can be effectively solved by spraying or preparing functional surfaces directly on the surface.

**Fig. 14:**
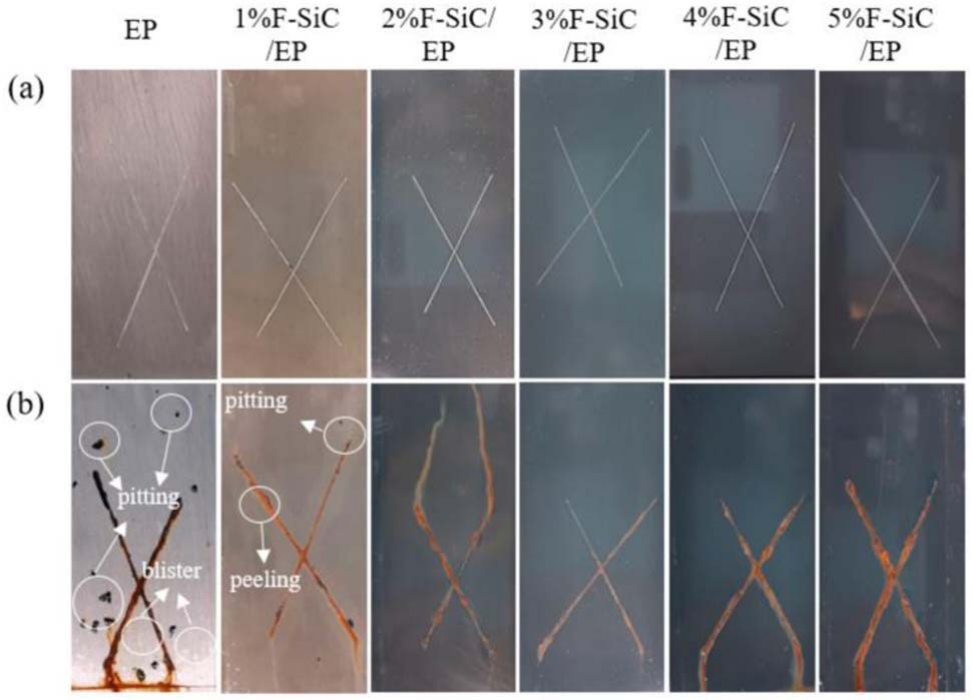
Photographs of different coating salt spray tests: (a) before the spray test; (b) 240 h after the salt spray test. Reproduced with permission.^52^ Copyright MDPI.

The preparation of superhydrophobic coatings utilizing nanoparticle-modified polymers requires the modification of nanoparticles with low surface energy to improve the agglomeration of the particles and at the same time increase the contact angle of the coatings. Zhang improved the hydrophobicity, abrasion resistance, and corrosion resistance of the material by the incorporation of fluorinated SiC as a reference component in an epoxy resin. Experiments were conducted to compare the reference components from 1 wt% to 5 wt%. The best overall performance was found with 3 wt%. Compared with the EP coating, the water contact angle of the coating increased by 62.9%, the coefficient of friction decreased by 73.5%, and the corrosion current decreased by three orders of magnitude (Fig. 14).^52^

With the advocacy for environmentally friendly materials, fluorine-free materials are receiving more and more attention from the academic community. Li prepared a fluorine-free multifunctional superhydrophobic coating with a corrosion current density reduction of more than two orders of magnitude and a protection efficiency of up to 98.3%. In addition, it has excellent flame retardant and self-extinguishing properties, water–oil separation, *etc.* It can be applied to various environments and has a wide range of application prospects in the future.^53^

### 4.3. Self-cleaning

The deposition of dust and soluble salts on the outer insulating surface can lead to serious degradation of the electrical properties of the dielectric surface. Superhydrophobic materials can utilize the ultra-high droplet contact angle of their surfaces to clean the insulating surfaces through the rolling down of droplets on the surfaces, restoring the electrical properties to a certain extent.

Takashi has newly designed superhydrophobic surfaces with photocatalytic self-cleaning properties by applying co-deposition technology of photoactive TiO_2_ and hydrophobic PTFE. The use of UV light and photoactive filler phases, including natural sunlight, opens the way for keeping superhydrophobic surfaces clean without complex systems, thus realizing energy-saving and maintenance-free self-cleaning superhydrophobic coatings.^54^

**Fig. 15:**
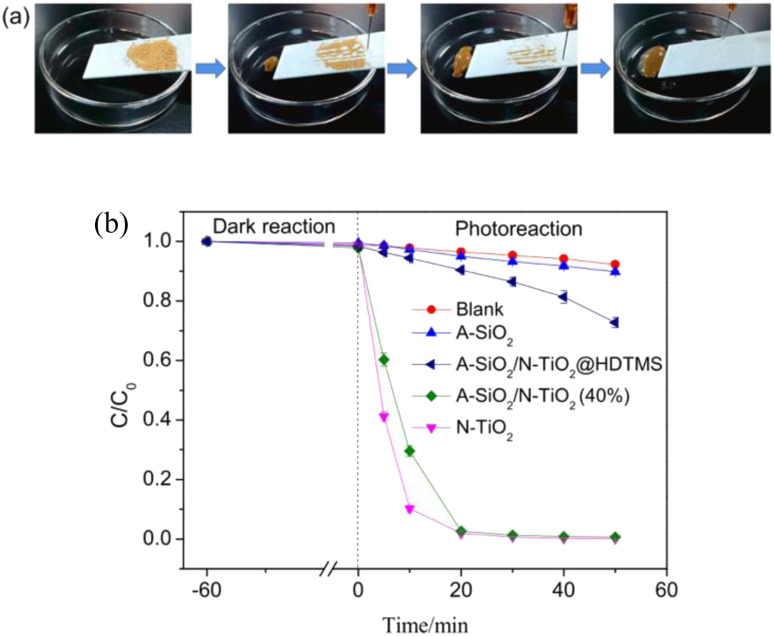
(a) Simulation experiment of the “lotus effect”; (b) photodegradation of methyl orange by A-SiO_2_/N-TiO_2_@HDTMS and other samples. Reproduced with permission.^55^ Copyright Elsevier.

Also utilizing the photocatalytic ability of TiO_2_, Wang constructed nano-TiO_2_ and SiO_2_ microsphere-encapsulated structures as filler phases, which were surface-modified and then sprayed on the surface of the substrate. The superhydrophobic self-cleaning coating with self-healing ability was prepared without using too many chemicals and step-by-step processes. This provides an idea for the large-scale application of this coating (Fig. 15).^55^

### 4.4. Anti-icing

The anti-icing principle of superhydrophobic materials is to reduce the amount of water collected at the interface and at the same time reduce the adhesion of the surface liquid and the heat transfer of the interface. On the one hand, it prevents the surface from icing. On the other hand, it makes it easier to de-ice the interface.

Guo prepared anti-ice surfaces by designing robust micro- and nano-scale structures. With this design, the micro- and nano-surfaces produce a longer delay time, about 7200 seconds, to stop ice formation at subzero temperatures of −10 °C. This finding is important for the design of novel anti-icing surfaces and provides a structural reference for longer-duration anti-icing materials.^56^

**Fig. 16:**
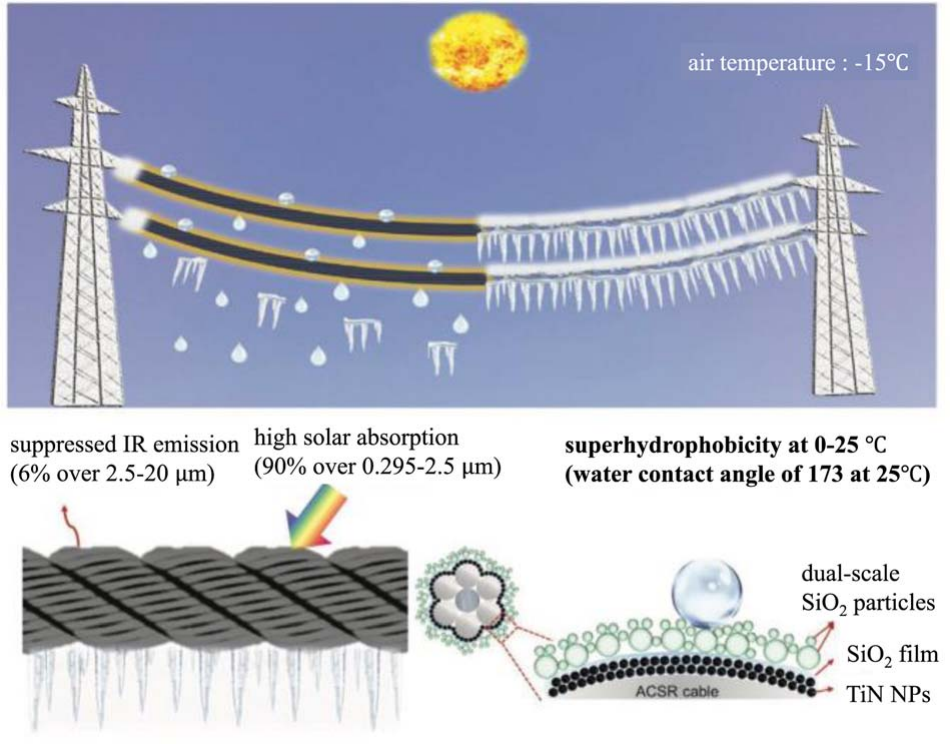
Schematic of the low-emissivity solar-assisted superhydrophobic (LE-SS) nanocoating for de-icing/defrosting on power transmission lines. The LE-SS coating can harvest and convert 90% of sunlight into heat but suppress the heat loss *via* thermal radiation due to the low emissivity of 6%. The LE-SS coating with dual-scale SiO_2_ particles is superhydrophobic with a water contact angle of 173°. Reproduced with permission.^57^ Copyright 2022 Wiley-VCH GmbH.

Li added titanium nitride nanoparticles as a solar-absorbing material to the double-scale structure. The solar energy absorption was up to 90% and the emissivity was only 6%. It can rise 72 °C in just one degree of solar radiation. The ice layer on the surface could be removed in 860 s at −15 °C. This adds active de-icing to the superhydrophobic coating on top of anti-icing (Fig. 16).^57^

With the development of various industries, problems in various fields have given rise to research and progress in functional media. The special wettability of superhydrophobic materials has led researchers to develop and optimize them while continuously discovering the value of superhydrophobic materials in various fields of application, such as self-cleaning, anti-fog, anti-oil, anti-corrosion, fluid drag reduction, and frictional power generation.

Electrified railways are the arteries of national economic development and their safe and stable operation is related to the steady advancement of national economic security. The long-term operation of electrified railways has revealed some long-standing problems: the accumulation of dirt on the outer insulation and lines; the threat of flashover of the outer insulation equipment caused by fog and ice coverage; the increased accumulation of dirt caused by oil pollution and the difficulty of removal; the bow network system is blocked by the flow due to the ice covering the contact network; the grounding end is subject to electrochemical corrosion; the further speed increase of the high-speed railway is limited by the surface resistance and other problems. The versatility of superhydrophobic materials makes them ideal candidates for solving these problems.

## 5. Conclusion & outlook

In recent years, the performance of biomimetic man-made superhydrophobic surfaces has gradually improved with the improvement of observation and preparation techniques. Inspired by nature, they have been applied in various fields through the continuous efforts of researchers. The versatility of superhydrophobic materials can be used in a wide range of applications, for example, in the field of electrified railways, where insulator fouling and line icing problems can be improved by the self-cleaning and anti-icing properties of superhydrophobic materials. When materials are applied in new fields, they are accompanied by new problems that need to be explored and tackled.

(1) Electrical, thermal, and mechanical properties of the material. The required roughness of superhydrophobic materials requires a high filling volume fraction. The shallow traps introduced lead to low flashover voltages along the surface; the poor thermal conductivity of the polymer itself causes thermal accumulation, accounting for 70% of electrical equipment failures; in engineering applications, substandard stability is also a common problem for superhydrophobic materials. A superhydrophobic material that is suitable for the surface of electrical equipment requires further modification of existing materials.

(2) Material preparation and coating methods. It should be determined which of the many methods of material preparation is the most cost-effective. Some surfaces in dielectrics are inherently hydrophobic, and whether such surfaces should be coated directly, indirectly, or by hot pressure (may destroy the perfect surface), sputtering, *etc.* needs further study.

(3) The economics and sustainability of the material. To achieve superhydrophobic properties, most materials are currently artificially fluorinated on the surface, which is not only harmful to the environment, causing the accumulation of fluorine in the environment and threatening biosafety, but also greatly increases the overall cost of the material. Finding a suitable surface modifier to achieve functionality, reduce cost, and at the same time achieve sustainability is an important development direction for the discipline of engineering materials.

## Conflicts of interest

There are no conflicts to declare.

## Supplementary Material
